# The expression and role of tyrosine kinase ETK/BMX in renal cell carcinoma

**DOI:** 10.1186/1756-9966-33-25

**Published:** 2014-03-07

**Authors:** Jintao Zhuang, Xiangan Tu, Kaiyuan Cao, Shengjie Guo, Xiaopeng Mao, Jincheng Pan, Bin Huang, Xu Chen, Yong Gao, Shaopeng Qiu

**Affiliations:** 1 Department of Urology, The First Affiliated Hospital, Sun Yat-sen University, Guangzhou 510080, China; 2 Research Center for Clinical Laboratory Standard, Zhongshan Medical School, Sun Yat-sen University, Guangzhou 510080, China; 3 Department of Urology, Sun Yat-sen University Cancer Center, Guangzhou 510060, China

**Keywords:** Renal cell carcinoma, ETK, Small interfering RNA

## Abstract

**Background:**

Expression of the non-receptor tyrosine kinase ETK/BMX has been reported in several solid tumors, but the underlying molecular mechanisms and its clinical significance in renal cell carcinoma (RCC) remain to be elucidated.

**Methods:**

ETK expression in 90 human RCC and 30 human normal renal tissue samples was examined by immunohistochemistry and compared with several clinicopathologic parameters. To further demonstrate the biological function of ETK in RCC, Western blot was used to test the expression level of ETK protein in RCC cell lines. Subsequent to the downregulation of ETK by small interfering RNA, the effects of ETK on RCC cell growth, apoptosis, migration and invasion were assessed by methyl thiazol tetrazolium assay, flow cytometry and transwell assay. And the varying expression of VEGF, STAT3 and phosphorylated STAT3 (p-STAT3) in RCC were evaluated by Western blot.

**Results:**

Immunohistochemistry analysis showed that ETK expression was highly increased in RCC and was positively correlated with clinical stage, grade and metastasis. Simultaneously, the overall survival time in patients with higher ETK expression was obviously shorter than that in patients with lower ETK expression. ETK was also detected in RCC cell lines. Moreover, the down-regulating ETK significantly inhibited RCC cell growth, migration, invasion and promoted apoptosis. The expression of VEGF and p-STAT3 were also decreased.

**Conclusions:**

Our study suggests that the overexpression of ETK is associated with the malignancy and disease progression of RCC. Since ETK is also involved in RCC cell biological function and VEGF-ETK-STAT3 loop, ETK may be used as a potential therapeutic target for RCC.

## Background

RCC is one of the most common malignant tumors in urology. RCC accounts for 2-3% of all malignant tumors in adults, afflicts about 209,000 people, and causes 102,000 deaths per year worldwide. The incidence and mortality rate of RCC have increased over the past several years [1,2]. RCC is classified into five major subtypes: clear cell (the most common type, which accounts for 82%), papillary, chromophobe, collecting duct and unclassified RCC. Many renal masses remain asymptomatic and nonpalpable until the late stages of the disease. Curative nephrectomy is the first treatment choice for RCC. However, metastatic disease recurs in a third of these patients. Still, About 30% of patients already have metastasis at the time of diagnosis [3]. Although several promising biomarkers for RCC such as Carbonic anhydrase IX, B7-H1 and P53 have been investigated, none have been validated [4,5]. RCC is resistant to chemotherapy, radiotherapy and immunotherapy. Although several targeted therapies, such as multitargeted tyrosine kinase inhibitors (TKIs) and Temsirolimus, which target the VHL-HIF-VEGF and/or mTOR pathways, have been approved for the treatment of advanced RCC, complete responses are rare and resistance ultimately will occur after a few months or a few years [6]. Thus, the identification and application of novel therapeutic targets for RCC are urgently needed.

Epithelial and endothelial tyrosine kinase (ETK), also known as bone marrow X kinase (BMX), is one member of the Tec family of non-receptor tyrosine kinases. ETK contains a PH (pleckstrin homology) domain, a SH3 (SRC homology 3) domain, a SH2 (SRC homology 2) domain from the amino terminus, and the kinase domain in the carboxyl terminus [7,8]. ETK is expressed in epithelial cells and distributed in lympho-haematopoietic cells [9]. ETK can be activated by several extracellular stimuli, including growth factors, cytokines, extracellular matrix and hormones [10]. ETK is a major regulatory molecule in various cell signal pathways, and therefore plays an important role in the initiation, transformation, progression and metastasis of cancer [11,12]. It has been proven that ETK is a critical mediator of Src-induced cell transformation and STAT3 activation. Src-ETK-STAT3 is an important pathway in cellular transformation [13]. However the expression and role of ETK in renal cell carcinoma still remain unclear.

In the present study, we demonstrated that ETK expression was upregulated in RCC tissue samples and cell lines. The overexpression of ETK was correlated with clinical stage, tumor grade, metastasis and survival time. Furthermore, ETK regulated cell proliferation, apoptosis, migration, and invasion of RCC. Our results suggest that ETK is a potential prognostic factor and may serve as a drug therapeutic target for RCC.

## Methods

### Tissue microarrays

Our tissue microarrays contain 90 specimens of RCC and 30 specimens of paracancerous normal renal tissues from the First Affiliated Hospital of Sun Yat-sen University (Guangzhou, China) between January 2005 and November 2011. All RCC patients were treated by radical nephrectomy. All samples were histologically confirmed. Among 90 RCC patients, there were 55 male and 35 female at a mean age of 55.2 years (ranging from 19 to 80 years). Tumors were staged according to the 2009 TNM staging system [14] and graded according to the criteria of the World Health Organization [15,16]. The Medical Ethics Committee of Sun Yat-sen University approved this study’s protocol.

### Cell culture

Five human RCC cell lines 786-O, 769-P, A-498, ACHN, OS-RC-2 and a normal renal proximal tubular cell line HK-2 were used in this study. 786-O, 769-P, ACHN and OS-RC-2 were purchased from the Cell Bank of the Chinese Academy of Sciences. A-498 and HK-2 were conserved in the lab of Research Center for Clinical Laboratory Standard of Sun Yat-sen University. 786-O, 769-P and OS-RC-2 were cultured in RPMI-1640 (Gibco, USA); A-498, ACHN and HK-2 were maintained in DMEM (Gibco, USA) containing 10% fetal bovine serum (Gibco, USA) at 37°C in a 5% CO_2_ atmosphere.

### Immunohistochemistry and evaluation of ETK expression

Tissue microarrays were deparaffinized with xylene and rehydrated through graded alcohol washes, followed by antigen retrieval by heating sections in sodium citrate buffer (10 mM, pH 6.0) for 10 min. The sections were incubated with 3% hydrogen peroxide for 10 min to block endogenous peroxidase activity at room temperature. Nonspecific reactions were blocked by incubating the sections in a solution containing normal goat serum (dilution 1:10). Then the slides were incubated with a 1:100 dilution of monoclonal mouse IgG anti-ETK antibody (BD, USA) at 4°C overnight. Following washing with PBS, slides were incubated with biotinylated secondary antibodies and avidin-biotin peroxidase complex (Dako Inc.) for 30 min. Reaction products were visualized by 3,3′-diaminobenzidine (DAB) and then counterstained with hematoxylin. The negative control was prepared by replacing the primary antibody with a primary antibody dilution buffer.

Using a microscope, two independent pathologists observed the distribution, staining intensity and positive ratio of ETK expression. The ETK immunohistochemical staining was classified as follows [17,18]: no staining scored 0; faint or moderate staining in ≤ 25% of tumor cells scored 1; moderate or strong staining in 25% to 50% of tumor cells scored 2; strong staining in ≥ 50% of tumor cells scored 3. For each sample, 4 randomly selected areas were observed under high magnification and 100 tumor cells in each area were counted to calculate the proportion of positive cells. Positively high expression of ETK was defined as staining index ≥ 2. Low expression of ETK was defined as staining index<2, accordingly.

### Western blot analysis

The expression of ETK in 786-O, 769-P, A-498, ACHN, OS-RC-2 and HK-2 cells was detected by Western blot as described previously [19]. Briefly, total proteins were extracted from RCC cell lines and denatured in a sodium dodecyl sulfate (SDS) sample buffer, then equally loaded onto 10% polyacrylamide gel. After electrophoresis, the proteins were transferred to a polyvinylidene difluoride membrane. Blots were incubated with the indicated primary antibodies overnight at 4°C and detected with horseradish peroxidase-conjugated secondary antibody (dilution 1:10,000, Abcam, USA). The mouse monoclonal anti-ETK antibody (BD, USA), the rabbit monoclonal anti-STAT3 antibody (Abcam, USA), the rabbit monoclonal anti-phospho-STAT3 antibody (Abcam, USA) and the rabbit monoclonal anti-VEGF antibody (Abcam, USA) were used at the dilution of 1:1,000, whereas anti-β-actin (Santa Cruz, USA) was used at the dilution of 1:2,000.

### RNA interference (RNAi) to knockdown ETK

We chose two typical clear cell RCC cell lines 786-O and 769-P for further study. As described in the literature [20,21], 786-O and 769-P cells were transfected with small interfering RNA (siRNA) against ETK and negative control siRNA by Lipofectamine 2000 and Opti-MEM I (Gibco, USA) according to the manufacturer’s protocol. All siRNAs were purchased from RiboBio Co. China, siRNA concentrations were 100 nM. Briefly, 1 × 10^5^ cells were plated in each well of 6-well plates and cultured to reach a 80% confluence. Cells were then transfected with siRNA by using the transfection reagent in serum-free medium. Total RNA and protein were isolated at 48 h after transfection. ETK expression was monitored by real-time reverse transcription-polymerase chain reaction (RT-PCR) and Western blot, as mentioned above.

### Real-time reverse transcription-polymerase chain reaction

For real-time RT-PCR, total RNA was isolated from 786-O and 769-P cells transfected with ETK siRNA or control siRNA using Trizol Reagent (Invitrogen, USA) as the manufacturer’s protocol required, and subjected to reverse transcription in 20 μl using reverse transcriptase of First Strand cDNA Synthesis Kit (Invitrogen, USA). RNA concentrations were 1–5 μg/μl. Then amplification was carried out in a total volume of 25 μl using SYBR Premix Ex Taq Kit (ABI, USA). The sequences of ETK primers were as follows: forward, 5′-GAGCCGAAGTCAGTGGTTGA-3′; reverse, 5′-ACTTCCCGTCCACGAAGAAC-3′. The sequences of internal control glyceraldehyde-3-phosphate dehydrogenase (GAPDH) were as follows: forward, 5′-TGTTCGTCATGGGTGTGAAC-3′; reverse, 5′-ATGGCATGGACTGTGGTCAT-3′. All PCR were performed in triplicate.

### Cell proliferation assay

3-(4,5-Dimethylthiazol-2-yl)-2,5-diphenyltetrazolium bromide (MTT) assays were performed by the following well-established method. In a 96-well plate, 1.0 × 10^4^ cells were plated in each well. The cells were incubated for 48 h. MTT was dissolved in phosphate-buffered saline (5 mg/ml) and filter-sterilized. Before the incubation, 20 μl of MTT solution was added to each well. The plate was incubated in an incubator at 37°C for 4 h. Media were aspirated gently, and 150 μl of dimethyl sulfoxide (DMSO) was added to each well to dissolve formazan crystals. The absorbance was measured at 490 nm. All experiments were performed in triplicate, and the cell proliferation was tested using the absorbance.

### Flow cytometry analysis for apoptosis

Detection of apoptosis by flow cytometry was performed using the Annexin V-FITC/PI Apoptosis Detection Kit (Roche, Switzerland). The transfected cells were harvested with trypsinization (no Ethylene Diamine Tetraacetic Acid, EDTA containing). Staining was performed according to the producer’s manual. Flow cytometry (BD, USA) was performed immediately.

### Migration and invasion assay

Cell migration and invasion were assessed using the 24 well-plate transwell insert (BD, USA) according to the manufacturer’s instructions. For cell migration, a transwell insert without matrigel was used; while for cell invasion, the transwell filters were pre-coated with matrigel [22]. In brief, 500 μl of prepared serum-free suspension of transfected cells with ETK siRNA or negative control siRNA (1 × 10^5^ cells/ml) was added into the interior of each insert (8 μm pore size); 500 μl of medium containing 10% fetal bovine serum was added to the lower chamber of the insert. Cells were incubated at 37°C in a 5% CO_2_ atmosphere for 36 h to 48 h. Then, non-invading cells in the interior of the insert were gently removed with a cotton-tipped swab; invasive cells on the lower surface of the inserts were stained with the staining solution for 20 min and counted under a microscope. All experiments were performed in triplicate.

### Statistical analysis

Statistical analysis was performed using SPSS 16.0 software. The relationship between ETK expression and the clinicopathologic features of RCC was assessed by chi-square test or Fisher’s exact test. Continuous data was analyzed by *t*-test or one-way analysis of variance (ANOVA) followed by Bonferroni’s post-hoc test. Survival curves were plotted using the Kaplan-Meier method and compared with the log-rank test. *P* value of less than 0.05 was considered statistically significant.

## Results

### ETK overexpression in RCC tissues and its relationship with the clinicopathological parameters

**Figure 1:**
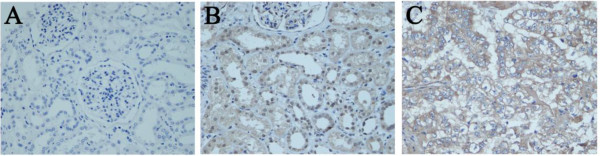
**Immunohistochemical staining of ETK protein in tissue microarray. A** Minimal expression of ETK in paracancerous normal renal tissue (×400), **B** Low expression of ETK in paracancerous normal renal tissue (×400), **C** High expression of ETK in RCC tissue (×400).

Immunochemical staining tests showed that ETK protein was mostly located in the cytoplasm as yellow-to-brown staining in the RCC tissues. ETK expression was weak in normal renal tissues, but stronger staining was observed in RCC tissues (Figure 1). As shown in Table 1, ETK protein was highly expressed in 56 of 90 (62.2%) primary RCC, while only expressed in 2 of 30 (6.7%) normal tissues. The difference was statistically significant (*P*<0.001). Furthermore, ETK expression was significantly correlated with clinical staging (*P* = 0.005), pathological grade (*P* = 0.001) and metastasis (*P* = 0.002). However, it was not associated with age (*P* = 0.788), gender (*P* = 0.322) or position of the tumor (*P* = 0.351). Taken together, these observations showed that high level of ETK expression were closely associated with the clinical progession of RCC.

**Table 1:** Correlation between ETK expression and the clinicopathological parameters of RCC

**Clinical data**	***n***	**ETK**		***P *** **value**
		**High expression**	**Low expression**	
		**(staining index ≥ 2)**	**(staining index<2)**	
Normal renal tissus	30	2	28	<0.001
RCC tissues	90	56	34	
Age (years)				0.788
≤50	28	16	12	
50~70	50	32	18	
≥70	12	8	4	
Gender				0.322
Male	55	32	23	
Female	35	24	11	
Position of the tumor				0.351
Upper pole	36	20	16	
Central part	19	11	8	
Lower pole	35	25	10	
TNM stage				0.005
I + II	67	36	31	
III + IV	23	20	3	
Grade (Differentiation level)				0.001
Well	66	34	32	
Moderately and poorly	24	22	2	
Metastasis				0.002
No	72	39	33	
Yes	18	17	1	

### Correlation of ETK expression with overall survival

**Figure 2:**
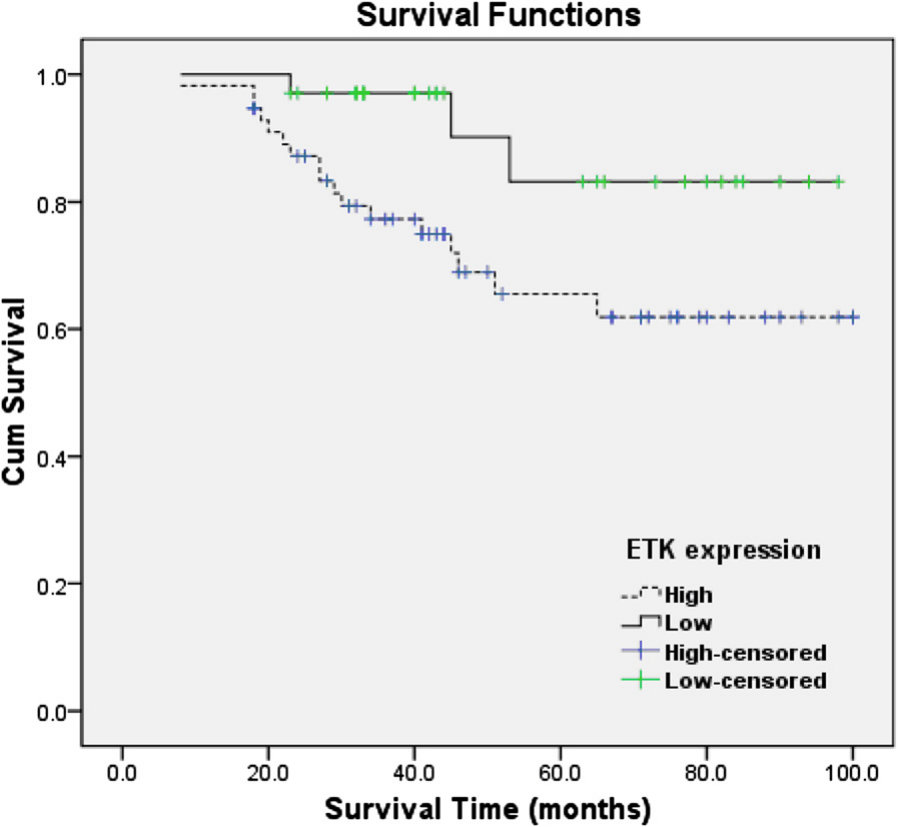
**Correlation of ETK expression with overall survival.** Kaplan-Meier curves with univariate analyses (log-rank) between RCC patients with high ETK expression (dotted line) and low ETK expression (thick line) (*P<0.05).

Clinical outcome analysis was performed on all of the 90 RCC patients underwent radical nephrectomy who were followed up for a median of 49.6 months. There were 56 tumors (62.2%) with high expression (ETK staining index ≥ 2) and 34 tumors (37.8%) with low expression (ETK staining index<2). Kaplan-Meier survival analysis indicated higher levels of ETK expression were associated with shorter survival time. Moreover, the log-rank test showed that overall survival was significantly different between the low and high ETK expression groups (*P*<0.05). As shown in Figure 2, the cumulative 5-year survival rate was 83.2% in the low-ETK-expression group, and 65.5% in the high-ETK-expression group.

### Upregulation of ETK in RCC cell lines

**Figure 3:**
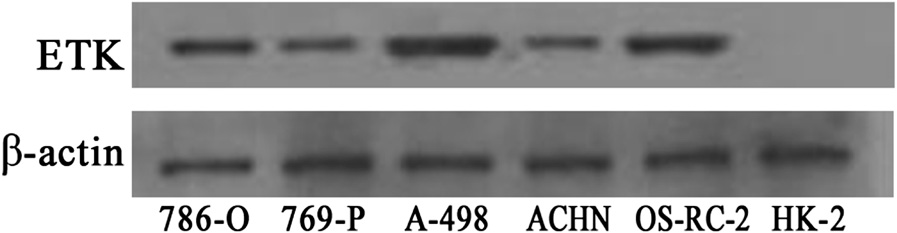
**Expression of ETK in RCC cell lines.** Western blot shows that ETK is highly expressed in all five RCC cell lines, but hardly expressed in the normal renal proximal tubular cell HK-2. β-actin is the loading control.

We detected the expression of ETK in five RCC cell lines (786-O, 769-P, A-498, ACHN, OS-RC-2) and a normal renal proximal tubular cell line HK-2 using Western blot. The results showed that ETK was highly expressed in all RCC cell lines, whereas it was hardly detected in the normal renal proximal tubular cell HK-2 (Figure 3).

### Effects of ETK on cell proliferation, apoptosis, migration and invasion

**Figure 4:**
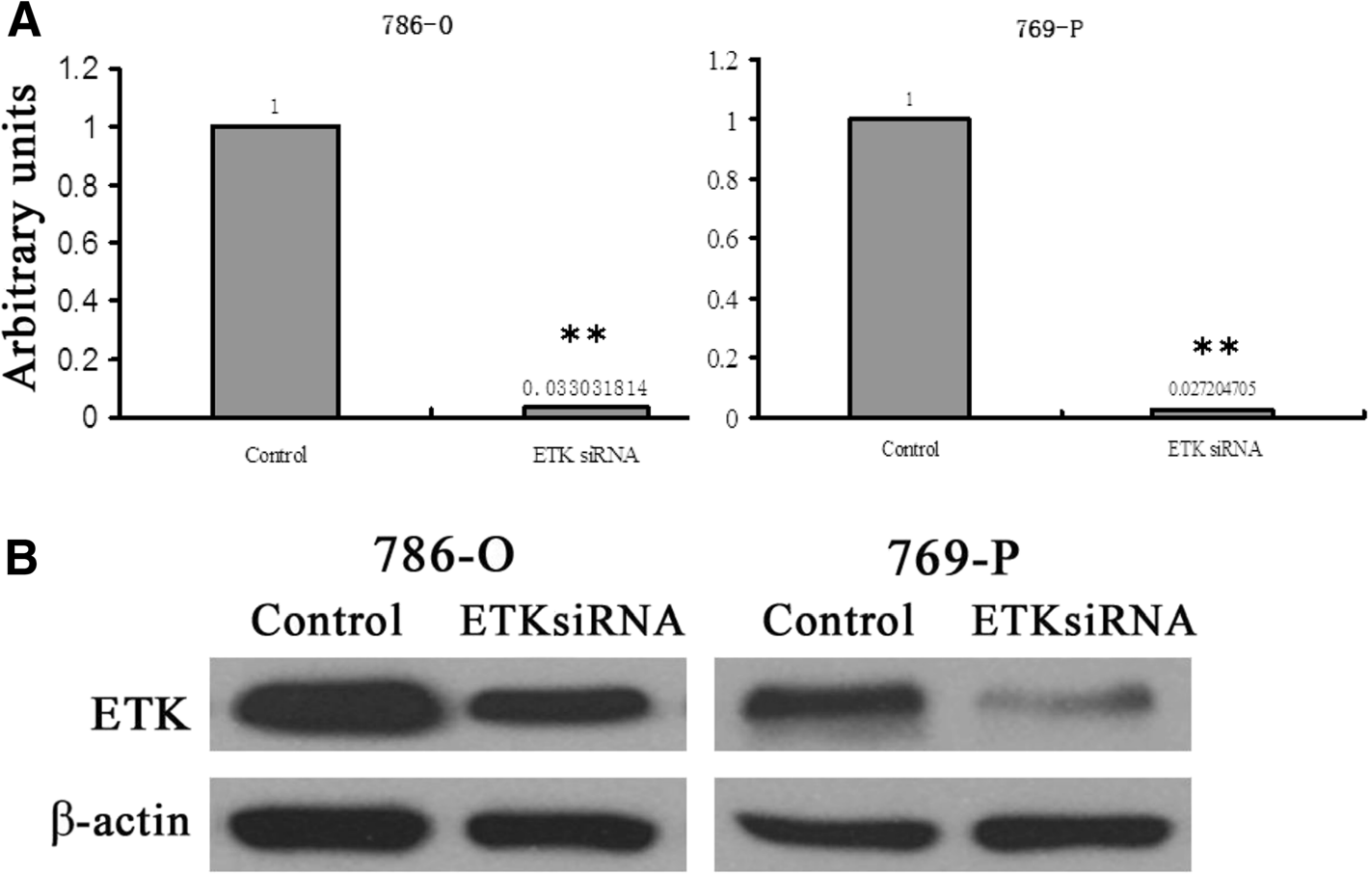
**Expression spectrum of ETK in ETK-siRNA-transfected and control-siRNA-tranfected RCC cells (786-O and 769-P). (A)** real-time PCR The average ratios of ETK mRNA expression in siRNA group and control group. Expression intensity of ETK was normalized by β-actin. Data were from three parallel experiments. **(B)** Western blot ETK expression in protein level in siRNA group and control group. Protein concentrations were 8 μg/ml. β-actin is the loading control. (**, *P*<0.01).

To examine the functions of ETK, we knocked down ETK by tranfecting ETK siRNA into RCC cells. We chose two typical clear cell RCC cell lines 786-O and 769-P for further study. The mRNA and protein expression of ETK were significantly weaker in ETK siRNA-transfected cells than that in control siRNA-tranfected cells (*P*<0.01) (Figure 4). For 786-O and 769-P respectively, the mRNA expression of ETK was decreased by 96.7% and 97.3% in the siRNA group compared with the negative control group (Figure 4A). Western blot showed that the expression level of ETK was decreased by 51.2% in 786-O and 79.8% in 769-P in the siRNA group compared with the negative control group (Figure 4B). These results suggested we have succeeded in knocking down ETK expression.

**Figure 5:**
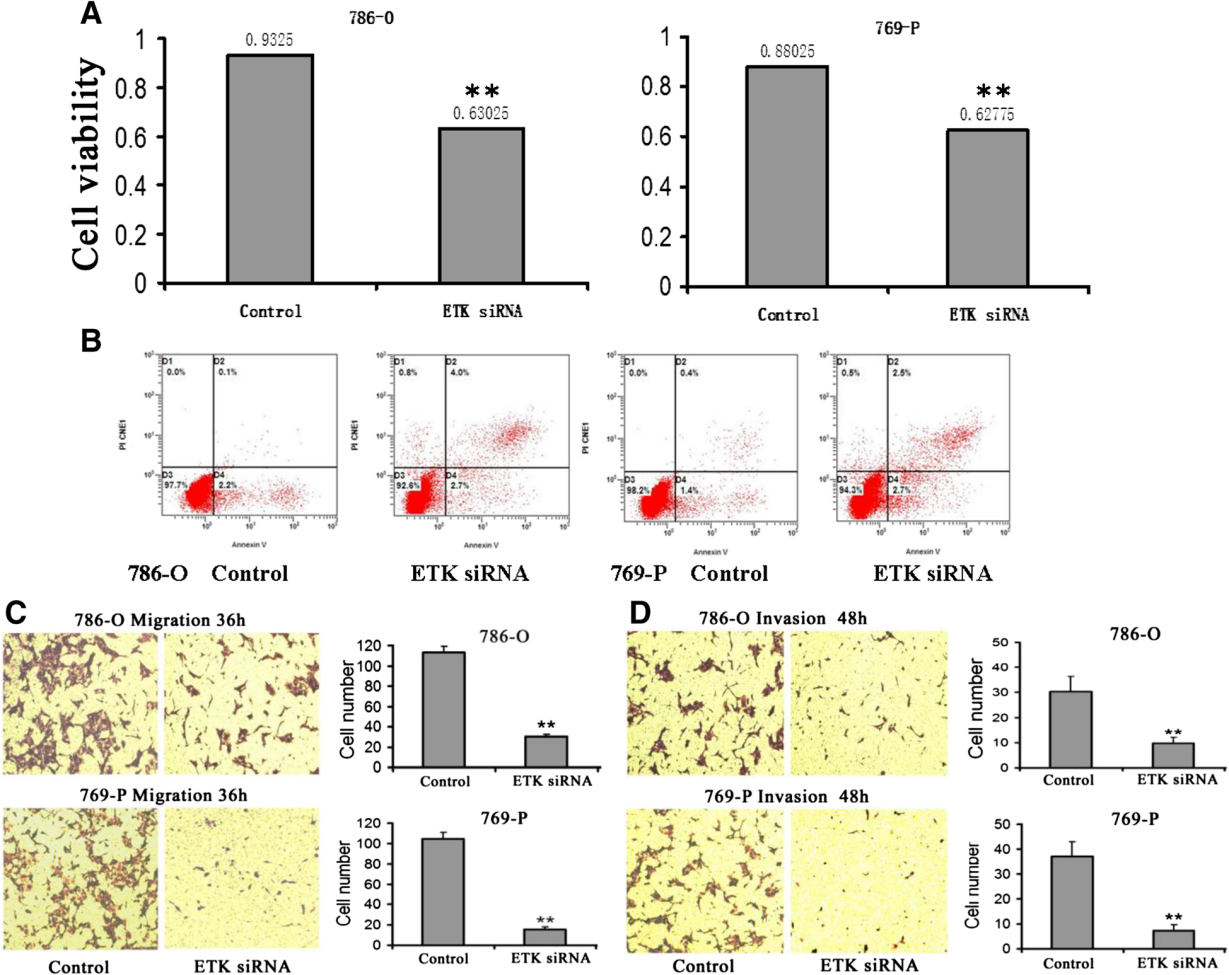
**Effects of ETK on cell proliferation, apoptosis, migration and invasion of RCC cells 786-O and 769-P.** Comparing ETK siRNA group with negative control siRNA group, **(A)** Cell proliferation was significantly inhibited after ETK siRNA transfection at 48 h. **(B)** Flow cytometry indicated that cell apoptosis increased after ETK siRNA transfection at 48 h. **(C)** Cell migration was significantly inhibited after ETK siRNA transfection at 36 h. **(D)** Transwell matrigel invasion assay showed a significant decrease of invaded cells after ETK siRNA transfection at 48 h. (**, *P*<0.01).

In order to detect the role of ETK in RCC cell proliferation, we examined the effect of ETK siRNA on RCC cell growth. ETK siRNA significantly decreased cell proliferation by 32.4% in 786-O and 28.9% in 769-P at 48 h compared with the negative control group (*P*<0.01) (Figure 5A). And we used flow cytometry to reveal the effect of ETK on RCC cell apoptosis. ETK siRNA can promote cell apoptosis (Figure 5B). We used transwell assay to assess cell migration and invasion. The number of migrating cells was significantly decreased in ETK siRNA group compared with control siRNA group (30.0 ± 3.7 vs. 113.8 ± 3.3 in 786-O and 15.8 ± 2.5 vs. 104.8 ± 6.0 in 769-P, respectively) (*P*<0.01) (Figure 5C). The number of invading cells was significantly decreased in ETK siRNA group compared with control siRNA group (9.8 ± 1.0 vs. 30.5 ± 2.1 in 786-O and 7.3 ± 1.3 vs. 37.0 ± 7.9 in 769-P, respectively) (*P*<0.01) (Figure 5D). Our data implied that ETK knockdown inhibited cell migration and invasion *in vitro*.

### ETK knockdown regulates VEGF and STAT3 expression in RCC

**Figure 6:**
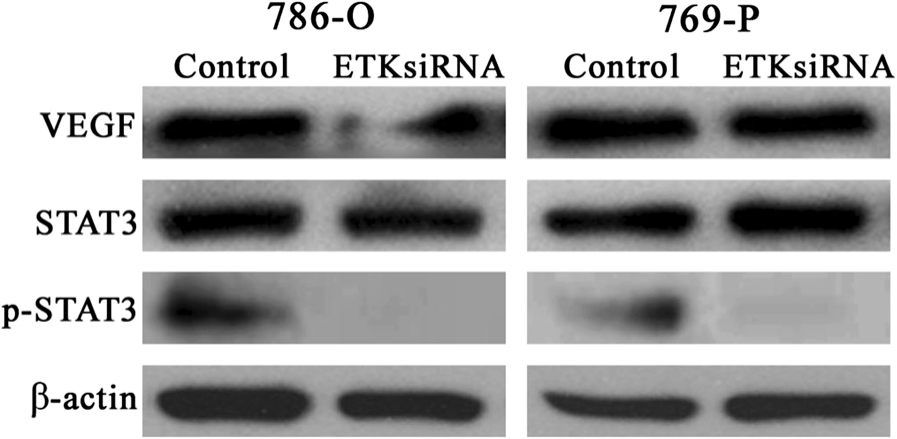
**Expression of VEGF and STAT3 after ETK knockdown.** Western blot showed that the expression of VEGF and p-STAT3 were decreased, especially the expression of p-STAT3 (*P*<0.01). While the unactivated STAT3 protein remained invariable (*P*>0.05). Protein concentrations were 8 μg/ml. β-actin is the loading control.

To explore the relationship between VEGF, STAT3 and ETK, we examined the expression of VEGF, STAT3 and p-STAT3 using Western blot after downregulating ETK. As shown in Figure 6, the expression of VEGF and p-STAT3 were decreased, especially the expression of p-STAT3 (*P*<0.01). The unactivated STAT3 protein meanwhile remained invariable (*P*>0.05). The expression of VEGF has changed but not of STAT3. Only STAT3s activity was altered as indicated by the expression of p-STAT3, whereas the expression of STAT3 remained unchanged.

## Discussion

In the recent few years, increasing evidences indicates that ETK is overexpressed in various cancer types, including prostate cancer, bladder cancer, nasopharyngeal carcinoma, lung cancer and breast cancer [18,22-25]. In this study, we evaluated the expression and role of ETK in RCC. Our results also showed that ETK was overexpressed in RCC tissues when compared with that in normal renal tissues. Furthermore, immunostaining data indicated that the expression level of ETK was closely correlated with clinical stage, histological grade and metastasis of the RCC. In addition, we also found that patients with higher ETK expression had shorter overall survival time than those with lower ETK expression. ETK may potentially be used as a prognostic factor for RCC patients.

ETK has been shown to regulate many cellular processes, including cell proliferation, apoptosis, migration, invasion, differentiation and chemo-resistance [26-28]. We found that ETK was highly expressed in all five RCC cell lines, whereas it was hardly detected in the normal renal proximal tubular cell HK-2. Frequently elevated ETK expression in RCC cells suggested that ETK may play a causal role in disease development and progression of RCC. We then adopted a strategy of RNA interference to inhibit ETK expression in two typical clear cell RCC cell lines 786-O and 769-P. Our results revealed that cell growth, migration and invasion were inhibited after transfection with ETK siRNA, and cell apoptosis increased instead. ETK is a major regulatory molecule in various cell signal pathways; multiple mechanisms are involved in ETK-regulated tumorigenesis. Experiments have documented that ETK overexpression can increase proliferation in mouse prostate epithelium and result in development of prostatic intraepithelial neoplasia (PIN) by increasing AKT and STAT3 activity [21,29]. ETK is an upstream activator of STAT family and links Src to STAT3 activation [13]. In addition, ETK can confer drug resistance by interacting with p53 and inhibiting its nuclear transduction function in prostate cancer [30]. It has been reported that ETK utilizes both MEK/ERK and PI3-K/Pak1 signaling pathways in concert to activate VEGF transcription. VEGF is both an ETK downstream target gene and an ETK upstream activator, constituting a reciprocal ETK-VEGF autoregulatory loop [31]. These mechanisms may explain the inhibited function of RCC cells by ETK knockdown in our study. As a result, we hypothesize the VEGF-ETK-STAT3 loop in RCC. Since ETK knockdown can regulate the expression of VEGF and STAT3 in RCC, ETK may play a key role in the VEGF-ETK-STAT3 loop which might be helpful to the theoretical treatment of RCC.

Like other cancer types, relapse and metastasis are the main causes of surgery failure in RCC treatment. RCC is resistant to chemotherapy, radiotherapy and immunotherapy. Patients with RCC respond to postoperative adjuvant therapy at various levels and usually cannot achieve expected outcomes [32]. For metastatic or non-resectable RCC, several targeted therapies, such as multitargeted tyrosine kinase inhibitors (TKIs) and Temsirolimus, have been approved for the treatment. They target the VHL-HIF-VEGF and/or mTOR pathways. Combination targeted therapy in advanced RCC is recommended. Even with improvements in survival, disease progresses in all patients. Resistance ultimately will occur after a few months or a few years [6,33]. Thus, the identification and application of novel therapeutic targets for RCC are urgently needed. The phenotype of tumor metastasis presents with promotion of cell proliferation, escape from apoptosis, and dysregulation of cellular adhesion and migration. The invasion of cancer cells to surrounding tissues and spreading to distal sites rely on cell migration ability [34]. In the present study, we found that ETK was highly expressed in about 90% of the advanced RCC patients. We stated that ETK expression was associated with high stage, bad differentiation level, and metastasis of RCC and higher levels of ETK expression were associated with shorter survival time. After silencing ETK by RNAi *in vitro*, the migration and invasion of RCC cells were significantly inhibited, suggesting that ETK may be one of the potential treatment targets for RCC.

## Conclusions

Our study indicated that the high expression of ETK could promote the carcinogenesis and progression of RCC and result in a poor overall survival. ETK may be involved in the VEGF-ETK-STAT3 loop and served as a potential therapeutic target for RCC, which warrants verification in further studies.

## Competing interests

The authors declare that they have no competing interests.

## Authors’ contributions

GSJ, MXP and PJC evaluated the immunostaining. CKY, HB and CX performed the statistical analysis. ZJT and QSP participated in the design of the study. ZJT and TXA drafted the manuscript. QSP and GY revised the manuscript. All authors read and approved the final manuscript.
